# Elevated myocardial fructose and sorbitol levels are associated with diastolic dysfunction in diabetic patients, and cardiomyocyte lipid inclusions in vitro

**DOI:** 10.1038/s41387-021-00150-7

**Published:** 2021-02-08

**Authors:** Lorna J. Daniels, Marco Annandale, Parisa Koutsifeli, Xun Li, Carol T. Bussey, Isabelle van Hout, Richard W. Bunton, Philip J. Davis, Sean Coffey, Rajesh Katare, Regis R. Lamberts, Lea M. D. Delbridge, Kimberley M. Mellor

**Affiliations:** 1 Department of Physiology, University of Auckland, Auckland, New Zealand; 2 Department of Physiology, HeartOtago, School of Biomedical Sciences, University of Otago, Dunedin, New Zealand; 3 Department of Cardiothoracic Surgery, Dunedin School of Medicine, University of Otago, Dunedin, New Zealand; 4 Department of Medicine and HeartOtago, Dunedin School of Medicine, University of Otago, Dunedin, New Zealand; 5 Department of Physiology, University of Melbourne, Melbourne, Australia; 6 Auckland Bioengineering Institute, University of Auckland, Auckland, New Zealand

**Keywords:** Cardiovascular diseases, Metabolism, Carbohydrates, Preclinical research

## Abstract

Diabetes is associated with cardiac metabolic disturbances and increased heart failure risk. Plasma fructose levels are elevated in diabetic patients. A direct role for fructose involvement in diabetic heart pathology has not been investigated. The goals of this study were to clinically evaluate links between myocardial fructose and sorbitol (a polyol pathway fructose precursor) levels with evidence of cardiac dysfunction, and to experimentally assess the cardiomyocyte mechanisms involved in mediating the metabolic effects of elevated fructose. Fructose and sorbitol levels were increased in right atrial appendage tissues of type 2 diabetic patients (2.8- and 1.5-fold increase respectively). Elevated cardiac fructose levels were confirmed in type 2 diabetic rats. Diastolic dysfunction (increased E/e’, echocardiography) was significantly correlated with cardiac sorbitol levels. Elevated myocardial mRNA expression of the fructose-specific transporter, *Glut5* (43% increase), and the key fructose-metabolizing enzyme, *Fructokinase-A* (50% increase) was observed in type 2 diabetic rats (Zucker diabetic fatty rat). In neonatal rat ventricular myocytes, fructose increased glycolytic capacity and cytosolic lipid inclusions (28% increase in lipid droplets/cell). This study provides the first evidence that elevated myocardial fructose and sorbitol are associated with diastolic dysfunction in diabetic patients. Experimental evidence suggests that fructose promotes the formation of cardiomyocyte cytosolic lipid inclusions, and may contribute to lipotoxicity in the diabetic heart.

## Introduction

Diabetes is associated with cardiomyocyte metabolic disturbances and cardiac dysfunction^1^. Population studies have associated diabetes and cardiovascular disease with escalating fructose consumption, but whether fructose plays a direct role remains controversial^2,3^. Experimentally, a high fructose diet induces cardiomyocyte metabolic and functional disturbances^4–7^. Although reported values of circulating fructose levels vary widely (5 µM–1.9 mM^8,9^), evidence suggests that plasma fructose is elevated in diabetic patients^10^. Myocardial tissue fructose levels in diabetic patients have not previously been investigated, and whether cardiomyocyte exposure to fructose contributes to cardiac pathology in diabetes is unknown. In type 1 diabetic rodent myocardium (streptozotocin-induced), upregulation of polyol pathway-mediated conversion of glucose to fructose has been demonstrated^11^. Thus, increased cardiomyocyte fructose levels in diabetes may derive from both circulating sources and endogenous production, but to date, rodent findings relating to polyol pathway disturbance have not been validated in the human context.

We have previously shown that the fructose-specific transporter GLUT5 is expressed in cardiomyocytes and provided evidence that fructose availability can modulate cardiomyocyte excitation-contraction coupling in vitro^12^. Additionally, fructose can directly impact structure and function of proteins via post-translational modifications which are both irreversible (advanced glycation end-products) and reversible (O-GlcNAcylation)^4,13^. The detrimental actions of fructose have been most well described in the liver, where increased fructose has been linked to lipid accumulation, ATP depletion, and insulin resistance^14^. Whilst pathologic impacts of fructose metabolism in the diabetic heart are yet to be characterized, some evidence of cardiac fructose involvement in heart failure progression in hypertrophic cardiomyopathy has been reported^15^.

The goals of this study were to characterize myocardial fructose levels in a clinical diabetic context, and to examine the cardiomyocyte metabolic consequences of fructose exposure using experimental approaches.

## Methods

Fructose and sorbitol content was measured in right atrial appendage tissue from non-diabetic (ND) and type 2 diabetic (T2D) patients undergoing coronary artery bypass graft surgery, and cardiac fructose content and expression of fructose metabolic enzymes were assessed in left ventricle tissue of male Zucker diabetic fatty (ZDF) rats aged 20 weeks. All experiments were approved by the University of Auckland and the University of Otago Human and Animal Ethics Committees. Neonatal rat ventricular cardiomyocytes (NRVMs) were isolated from Sprague Dawley rats at days 1–2 and cultured in fructose (1 mM) or control (1 mM mannitol) conditions for 24 h prior to analysis for lipid inclusions (Oil Red O), protein expression (western blot) or metabolism (Seahorse XFp Bioanalyzer). Detailed methods are available in the online supplement.

## Results

### Increased cardiac fructose and sorbitol levels associated with diastolic dysfunction in diabetic patients

**Fig. 1:**
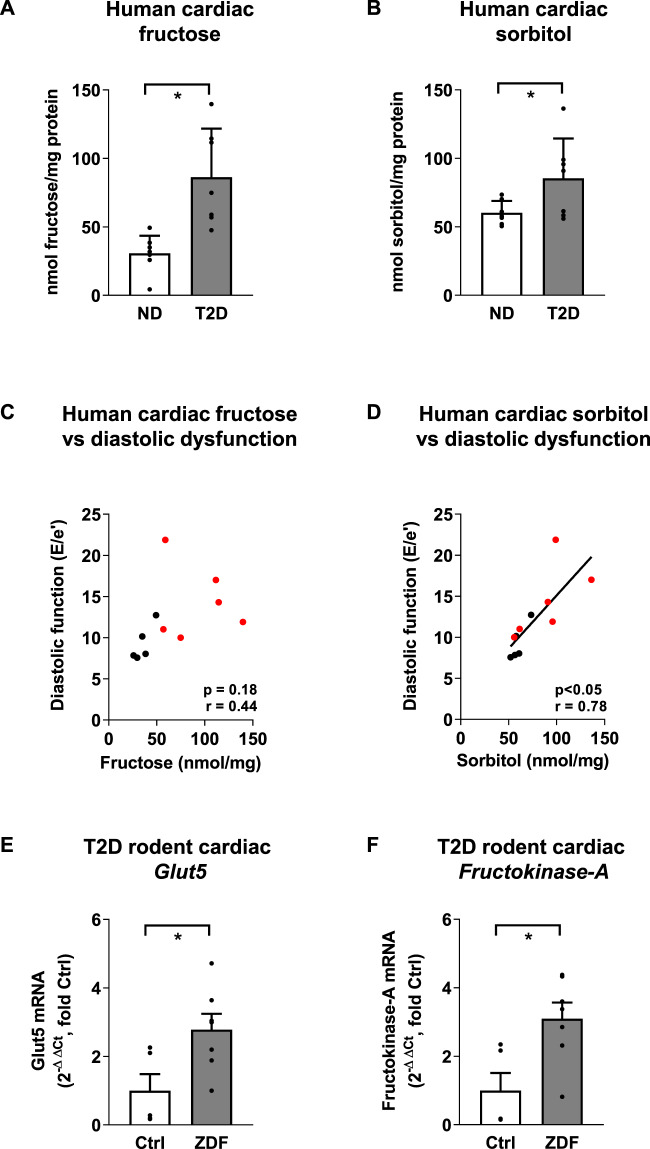
Myocardial fructose metabolism in type 2 diabetes. **A**, **B** Cardiac fructose and sorbitol levels are increased in human type 2 diabetic (T2D, *n* = 7) patient right atrial appendage samples relative to non-diabetic (ND, *n* = 8) patients. Mean ± SD. **C** Correlation of cardiac fructose levels and diastolic dysfunction (E/e’ ratio) in ND (black, *n* = 5) and T2D (red, *n* = 6) patients (*r*, Pearson correlation coefficient). **D** A significant positive correlation is evident between cardiac sorbitol levels and diastolic dysfunction (E/e’ ratio) for ND (black, *n* = 5) and diabetic (red, *n* = 6) patients (*r*, Pearson correlation coefficient). **E**, **F** *Glut5* and *Fructokinase-A* mRNA expression is increased in T2D rat hearts (Zucker diabetic fatty rats (ZDF), *n* = 7; Controls, *n* = 5). Non-parametric Mann Whitney U test **A**, **E**, **F**, unpaired Students t-test (**B**), *p-value < 0.05, mean ± SEM.

Non-diabetic and type 2 diabetic patients were clinically characterized, as shown in Table S1. Pre-surgical echocardiography revealed that left ventricular ejection fraction was preserved in both groups (T2D 56.0 ± 4.6% vs. ND 57.4 ± 5.8%). T2D patients exhibited diastolic dysfunction, evidenced by increased E/e’ (Table S1). Both cardiac fructose and sorbitol (polyol pathway intermediate) were markedly increased in right atrial appendage tissue from T2D patients (Fig. 1A, B, 2.8- and 1.5-fold increase respectively, T2D vs. ND, *P* < 0.05). Cardiac sorbitol levels and diastolic dysfunction were positively correlated (sorbitol vs. E/e’, Fig. 1D, *r* = 0.78, *P* < 0.05), suggesting involvement of endogenous cardiac fructose production in diastolic functional disturbance in T2D.

### Diabetic upregulation of expression of fructose-related genes in rat myocardium

Gene expression of the fructose-specific transporter, *Glut5*, and fructose metabolizing enzyme, *Fructokinase-A* (also termed *ketohexokinase-A*, phosphorylates fructose to fructose-1-phosphate), were evaluated in T2D rat hearts (Zucker Diabetic Fatty (ZDF) rat). Increased cardiac fructose levels were confirmed in ZDF rats (Fig. S1). Increases in mRNA expression levels of both *Glut5* and *Fructokinase-A* were detected (Fig. 1E, F, 2.7- and 3.1-fold increase respectively, ZDF vs. control, *P* < 0.05). These findings suggest that cardiac fructose transport and metabolism are increased in diabetes.

### Experimental evidence of fructose-derived cardiomyocyte glycolytic flux in vitro

**Fig. 2:**
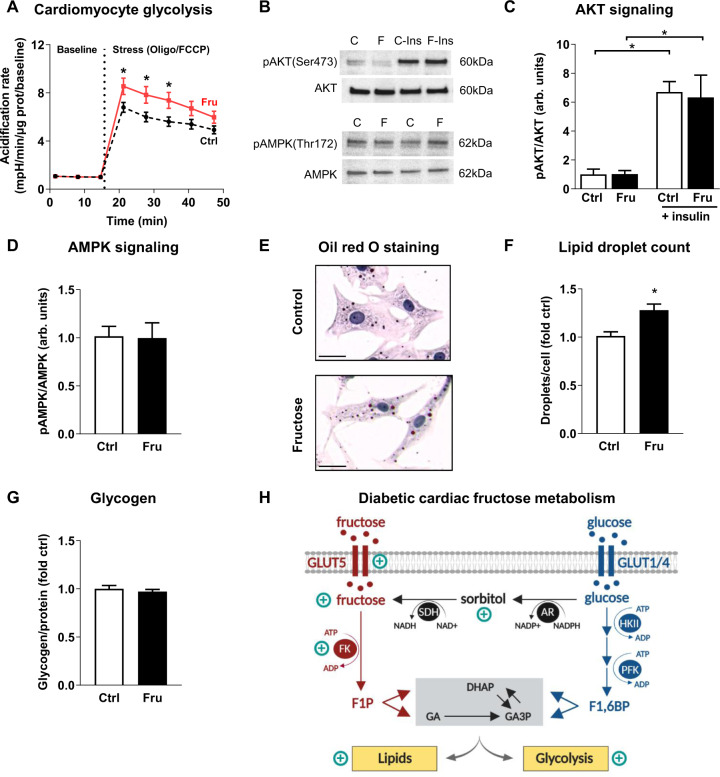
Effect of fructose on cardiomyocyte glycolysis, energy signaling, lipid droplets, and glycogen content in vitro. Neonatal rat ventricular myocytes were cultured for 24 h with 1 mM fructose (Fru) vs. control (Ctrl, 1 mM mannitol). **A** Increased glycolytic capacity with fructose was evident during the stress test (extracellular acidification rate normalized to protein & baseline). Oligo, oligomycin; FCCP, carbonyl cyanide-p-trifluoromethoxyphenylhydrazone. *n* = 9 wells/group, 3 independent cultures. **B** Representative immunoblots for **C**, **D** (C, control; F, fructose; Ins, insulin). Images have been cropped for concise presentation. **C** Ratio of phosphorylated (Ser473) to total AKT was unchanged with fructose in baseline and insulin-stimulated conditions (1 µm insulin, 30 min). **D** Ratio of phosphorylated (Thr172) to total AMPK was unchanged with fructose. **E** Representative images of control and fructose cultured cardiomyocytes, oil red O-stained for assessment of lipids (high-intensity red lipid droplets show as near black in some instances, scale bar 20 µm). **F** Cardiomyocyte lipid droplet count normalized to cell number (estimated by nuclei count), is increased with fructose (*n* = 5–6 wells/group, 10–15 images per well, 2 independent cultures). **G** Glycogen content was similar in cardiomyocytes cultured in fructose and control media (*n* = 7–11 wells/group, 3 independent cultures). **H** Schematic representation of the cardiomyocyte consequences of high fructose levels in the diabetic heart. FK, fructokinase (also termed ketohexohinase); F1P, fructose-1-phosphate; DHAP, dihydroxyacetone phosphate; GA, glyceraldehyde; GA3P, glyceraldehyde-3-phosphate; HKII, hexokinase II; PFK, phosphofructokinase; F1,6BP, fructose-1,6-biphosphate; AR, aldose reductase; NADPH, reduced form of nicotinamide adenine dinucleotide phosphate (NADP + ); SDH, sorbitol dehydrogenase; NADH, reduced form of nicotinamide adenine dinucleotide (NAD). Schematic created with biorender.com. Repeated measures ANOVA with Bonferroni post-hoc tests (**A**), 2-way ANOVA with Bonferroni post-hoc tests (**C**), Mann Whitney U non-parametric test (**F**), Unpaired Students *t*-test (**D**, **G**), *p-value < 0.05, mean ± SEM.

To obtain information regarding the involvement of glycolytic and mitochondrial metabolism in a more controlled setting, responses of NRVMs cultured in elevated fructose or mannitol (osmotic control) were investigated. In early development, cardiomyocyte glycolysis demand is accentuated, and NRVMs provide an optimal model system for evaluating hexose sugar metabolic impacts^15^. No difference in glycolysis between fructose and control incubated cardiomyocytes (extracellular acidification rate (ECAR)) was observed at baseline (Fig. 2A, fructose 1.0 ± 0.1 vs. control 1.1 ± 0.1 mpH/min/µg protein). In response to FCCP-Oligomycin induced depletion of mitochondrial-derived ATP, glycolysis (ECAR) increased to a greater extent in cardiomyocytes exposed to fructose than control (Fig. 2A, fructose: 8.5-fold increase, control: 6.8-fold increase, *P* < 0.05). No change in mitochondrial respiration was observed (Fig. S2A). Cardiomyocyte metabolic signaling via the canonical energy sensor pathways was unaffected by fructose exposure, indicated by no change in AKT and AMPK phosphorylation (Fig. 2B–D, Fig. S3). Collectively, these data suggest that fructose influence on glycolytic metabolism is consistent with direct fructose utilization as a glycolytic substrate, rather than secondary to altered energy signaling.

### Fructose increased cardiomyocyte lipid content but not glycogen content in vitro

Lipid accumulation is a key feature of diabetic cardiomyopathy and studies in non-cardiac cells have demonstrated that both lipids and glycogen can be derived from fructose^16,17^. NRVM histology showed that fructose exposure significantly increased cardiomyocyte cytosolic lipid droplet count (Fig. 2E, F, 28% increase, fructose vs. control, *P* < 0.05). This finding was confirmed in H9c2 rat cardiomyoblast cell line using spectrophotometric quantification of Nile Red lipid droplet staining (Fig. S2B). In contrast, fructose exposure did not influence glycogen stores in NRVMs (Fig. 2G).

## Discussion

This study is the first to demonstrate a clinical link between elevated myocardial fructose and sorbitol levels, and cardiac dysfunction in diabetes. Experimental exploration of the mechanisms involved revealed that myocardial tissues of a T2D rodent model exhibited upregulated gene expression of the *Glut5* fructose transporter and the fructose-phosphorylating enzyme, *Fructokinase-A*. In vitro analyses showed that fructose exposure exerts direct cardiomyocyte metabolic influence, conferring increased glycolytic capacity associated with cardiomyocyte cytosolic lipid droplet accumulation. Collectively these findings indicate a pathogenic role for elevated fructose and sorbitol in diabetic heart disease.

### Pathophysiology of endogenous fructose and sorbitol elevation in the diabetic heart

The extent of myocardial fructose elevation in the T2D diabetic patient cohort reported here is substantial, and demonstrates the clinical relevance of deranged fructose handling. In this heterogenous cohort of patients with limited sample size and variable medication status, a dramatic 2.8 fold increase in cardiac fructose content was observed. Previous reports of elevated myocardial fructose have been limited to rodent studies of pharmacologically induced or genetically produced diabetic conditions^11,18^. Our data show that cardiac fructose and sorbitol elevation occur in parallel, indicative of altered polyol pathway flux (i.e., conversion of glucose to sorbitol to fructose, Fig. 2H). Increased production of sorbitol via aldose reductase has been linked to cellular oxidative stress^19^ and advanced glycation end-product formation (methylglyoxyl)^20^. Clinical findings show that pharmacologic inhibition of the polyol pathway improves cardiac systolic function in diabetes^21^. Interestingly, our study found a significant correlation between cardiac sorbitol and diastolic dysfunction (increased E/e’) suggesting that upregulation of the polyol pathway intermediate (sorbitol) may have a specific pathophysiologic link relatively early in the progression of diabetic cardiomyopathy, even while systolic function is maintained. Diastolic dysfunction in diabetes is highly prevalent and the underlying mechanisms are not well understood. Potential contributors include increased cell death with fibrotic infiltration^22,23^, increased cardiomyocyte stiffness, and metabolic disturbance^1^.

Further work is required to understand the metabolic drivers of sorbitol elevation in the diabetic myocardium. Investigation into the influence of anti-diabetic medications on cardiac fructose uptake and/or production would be informative, and with large datasets it may be possible to systematically evaluate these possibilities. In a setting where cardiomyocyte glucose influx is restricted (by insulin insufficiency or resistance), sorbitol production directly from incoming glucose would be expected to be limited. A more complex metabolic derangement such as disruption in the localization of enzymatic complexes involved in glycolysis may favor sorbitol production in the diabetic cardiomyocyte.

### Diabetes-induced upregulation of cardiac fructose-related genes

Sorbitol increase in the diabetic cardiomyocyte may also be a kinetic consequence of elevated trans-sarcolemmal GLUT5-mediated fructose influx suppressing sorbitol dehydrogenase activity. In the present study, increased cardiac mRNA expression of the fructose-specific transporter, *Glut5*, was evident in a setting of elevated cardiac fructose levels in T2D rat hearts. GLUT5 exhibits high specificity for fructose, with negligible capacity to transport glucose^24^. Thus GLUT5-mediated fructose transport is realistic even in a context of elevated blood glucose (e.g., diabetes). Given that GLUT5 expression can be positively regulated by its substrate^24^, it is feasible that increased cardiac fructose in diabetes may trigger upregulation of GLUT5 expression, and the extent of cardiomyocyte fructose influx or efflux may be determined by the (as yet undefined) sarcolemmal fructose concentration gradient. *Fructokinase-A* mRNA expression, the enzyme that catalyzes the conversion of fructose to fructose-1-phosphate, was also increased in rodent T2D hearts, consistent with an increased capacity for cardiomyocyte fructose metabolism in diabetes. Future studies using isotopic labeling of fructose may be informative for understanding the metabolic fate of fructose in the heart.

### Metabolic consequences of elevated cardiomyocyte fructose

Previously, using adult cardiomyocytes in vitro, we demonstrated that fructose can be utilized as a fuel source, supporting contractility and Ca^2+^ handling^12^. In the present study, we have shown that cardiomyocyte fructose exposure increases glycolytic capacity without affecting key insulin and AMPK regulatory signals. This indicates that increased glycolytic capacity is related to the direct utilization of fructose as a glycolytic substrate. The effect of fructose on glycolysis was only evident under conditions of mitochondrial ATP inhibition (oligomycin), suggesting that fructose could be a potential glycolytic substrate in settings where aerobic metabolism is limited (e.g., hypoxia/ischemia), but is unlikely to contribute to glycolytic flux in ATP replete states. As fructose bypasses the glycolytic rate-limiting enzyme, phosphofructokinase, its metabolism can proceed in a relatively unregulated manner with the potential to increase lactate production and induce cellular acidosis. Whether fructose-derived glycolysis affects cellular ATP production, and can confer benefit or detriment in diabetic cardiomyocytes is yet to be determined.

In the present study, primary rat cardiomyocytes cultured in fructose exhibited an increase in the occurrence of cytosolic lipid inclusions, with no change in glycogen content. It can be speculated that cardiomyocyte fructose exposure may elicit lipid accumulation via direct production of lipids using fructose as a substrate (de novo lipogenesis or glyceroneogenesis) and/or via a fructose-induced signaling response promoting lipid production or inhibiting lipid breakdown. Given that myocardial lipid content has been shown to be an independent predictor of diastolic dysfunction in type 2 diabetic patients, fructose-induced lipids may have important implications for diabetic cardiomyopathy^25^. Further characterization of the cardiomyocyte metabolic fate of fructose is now required to probe the detailed mechanisms of fructose-associated cardiomyocyte lipid storage increase, potentially inducing lipotoxicity.

## Conclusion

In conclusion, this is the first study to identify that myocardial fructose and sorbitol are elevated in T2D patients, and to demonstrate that elevated intracellular fructose production (polyol pathway) may be linked to clinically-detected diastolic dysfunction. In vitro mechanistic evidence suggests that fructose promotes increased cardiomyocyte cytosolic lipid inclusions. Taken together these in vivo and in vitro studies provide a compelling case for cardiac fructose involvement in cellular lipotoxicity and metabolic dysregulation in diabetic cardiomyopathy. These findings provide the impetus for more extensive interrogation of cardiac fructose and sorbitol metabolism in the etiology of diabetic cardiomyopathy.

## Supplementary information

Supplementary materials and methods

Supplementary Figures and Table
