# Supplementary material for: Elevated myocardial fructose and sorbitol levels are associated with diastolic dysfunction in diabetic patients, and cardiomyocyte lipid inclusions in vitro
Source: Nutr Diabetes. 2021 Feb 8;11:8. doi: 10.1038/s41387-021-00150-7 (PMC7870957; doi:10.1038/s41387-021-00150-7)
Supplement: Supplementary file 1 — Supplementary materials and methods [file 41387_2021_150_MOESM1_ESM.docx]

**Supplementary materials and methods**

**Ethics.** Informed consent practices conformed to the principles outlined in the Declaration of Helsinki and was approved by the Human and Disability Ethics Committee of New Zealand (LRS/12/01/001). All animal experiments were approved by the University of Auckland and University of Otago Animal Experimentation Ethics Committees.

**Diabetic cardiac tissue collection.** Human right atrial appendage (RAA) tissue was collected from non-diabetic (ND, n=8) and type 2 diabetic (T2D, n=7) patients undergoing on-pump coronary artery bypass graft surgery. The T2D patient cohort had diabetes for >10 years with average blood glucose 9.6 ± 0.7 mM and HbA1c 56 ± 4.0 mmol/mol. Clinical echocardiographic examinations were performed 24 – 48 hours prior to surgery, as previously described [1]. Left ventricle tissue was dissected from 20 week old male Zucker diabetic fatty rats (ZDF homozygote *fa/fa*; n=5) and their non-diabetic littermates (wildtype, n=7), unblinded. Animals were obtained from a colony maintained at the University of Otago, New Zealand, derived from original breeding pairs sourced from Charles River Laboratories (Wilmington, MA, USA). Animals had ad libitum access to food and water. ZDF rats are a well-established model of T2D induced by a homozygous missense mutation (*fatty, fa*) in the leptin receptor gene (*Lepr*). ZDF rats develop T2D from 7 weeks of age characterized by hyperinsulinemia, hyperglycemia and increased body weight [2]. Frozen cardiac tissue was homogenized in 100 mM Tris-HCl, 5 mM EGTA, and 5 mM EDTA (Sigma-Aldrich) buffer containing protease and phosphatase inhibitors (Roche, Switzerland) for biochemical analysis, or homogenized in TRIzol for RNA extraction.

**Cell culture of neonatal rat ventricular cardiomyocytes.** Neonatal rat ventricular cardiomyocytes (NRVMs, male/female) were isolated from 1-2 day old Sprague Dawley rats as previously described [3]. Once established, NRVMs were cultured in serum-free Dulbecco’s modified essential media (DMEM; Sigma) containing 5 mM glucose, 1 nM insulin, and randomized to wells supplemented with mannitol-control (Ctrl: 1 mM mannitol) or fructose (Fru: 1 mM fructose) for 24 hours. Literature values of circulating fructose vary widely (~5 µM to 1.9 mM), likely dependent on the methodology employed. Thus 1 mM fructose was selected for these experiments as it falls within the upper-end of the range of reported values. Cells were either used for bioanalyzer experiments, or lysed with radioimmunoprecipitation buffer (RIPA, ThermoFisher) with protease and phosphatase inhibitors (Roche) for molecular analysis.

**Cell culture of H9c2 cardiomyoblast cell line with Nile Red lipid assay.**

H9c2 rat cardiomyoblast cells (Merck-Millipore) were cultured in DMEM supplemented with 25mM glucose and 10 % neonatal calf bovine serum (NCBD; Invitrogen). When confluence reached 90%, cell media was supplemented with mannitol-control or 1mM fructose for 24 hours. Intracellular lipid content was measured with Nile Red (Abcam, ab228553) as per manufacturer’s instructions. Briefly, cells were washed twice with PBS and were incubated in Nile Red staining solution at 37 °C for 30 minutes prior to measuring fluorescence intensity using a microplate reader (PerkinElmer EnSpire® Multimode Plate Reader) at Ex/Em = 550/640nm. Nile Red fluorescence was normalized to DAPI nuclear staining (Ex/Em = 348/478nm).

**Biochemical assays.** Fructose and sorbitol levels were assessed in human cardiac tissue homogenate (Abcam, ab83380, ab118968). Glycogen content was determined by enzymatic assay in NRVM lysates as previously described [3].

**qPCR mRNA analysis.**  RNA was extracted, reverse transcribed and gene expression evaluated by qPCR as previously described [4]. The primer pairs used were: rat *Fructokinase-A* (also termed *Ketohexokinase-A*), 5’-GCCATTCTGTGGACTTACG-3’ (forward) and 5’‑TTGAGAAGGTCGA-TCTGACC-3’ (reverse); rat *Slc2a5* (*Glut5*), 5’‑CAGCTGCTGAGAAAGCCCTTCAG-3’ (forward) and 5’‑ATCTCCACGATCGTCCTCATGGC-3’ (reverse).

**Bioenergetics analysis.** Glycolytic metabolism and mitochondrial respiration were assessed using the XFp Seahorse Bioanalyzer Cell Energy Phenotype Assay Kit according to manufacturer instructions (Agilent Technologies). Briefly, 1x10^5^ NRVMs per well were seeded in 8-well plates and cultured as per the control and fructose conditions described above. Following 24 hours of mannitol (osmotic control) or fructose experimental media, the cells were incubated in XFp base media (Agilent Technologies) supplemented with 5 mM glucose, 1 mM pyruvate, 4 mM glutamine, and 1 mM mannitol (Ctrl) or 1 mM fructose (Fru) in a CO_2_-free 37°C incubator (1 hr). The plate was then assayed to measure basal glycolytic rate (extracellular acidification rate, ECAR) and mitochondrial metabolism (oxygen consumption rate). Oligomycin (Oligo, 1 µM; ATP synthase inhibitor) and carbonyl cyanide-p-trifluoromethoxyphenylhydrazone (FCCP, 1 µM; mitochondrial uncoupler) were used to increase ATP demand and induce a maximum metabolic stressed state for measurement of glycolytic and mitochondrial capacity. After assays were performed, cells were lysed using RIPA buffer and protein content measured via Lowry assay. Data were analyzed using the Seahorse XFp Wave software and XF Cell Energy Phenotype Test Report Generator. Background adjustment and normalization of ECAR and OCR to protein concentration was performed.

**Protein expression.** Cell lysate protein expression was evaluated by immunoblot as previously described [3]. Equal protein amounts were loaded into SDS-PAGE gels and transferred to an immunoblot membranes which were probed with antibodies (Cell Signaling Technology) for phosphorylated AKT (Ser473; #9271), AKT (#9272), phosphorylated AMPK (Thr 172; #2535) and AMPK (#2531) and blots were quantitated densitometrically (Fig. S2). The phosphorylation sites of Akt (Ser473) and AMPK (Thr172) were selected for analysis as they are key determinants of the activation of these proteins.

**Histological analysis of myocyte lipids.** NRVMs were fixed (4% paraformaldehyde) and incubated with Oil Red O-isopropanol solution (Sigma-Aldrich), counterstained with Mayer’s hematoxylin and DPX mounted. Stained slides were imaged using a Zeiss Axio Imager Z2 microscope with Meta Systems VSlide scanner. Images were extracted using the VSlide Software at 20x magnification (15 images per slide, 600 x 300 µm). A customized Image J macro was used for automated lipid analysis. Briefly, red, green and blue color channels were separated and background was subtracted. The red and green channels were combined to enhance the contrast of the cellular regions. An ImageJ auto ‘minimum’ threshold was applied to identify and exclude the non-cell areas of the region of interest. For identification of lipid droplets, an ImageJ auto ‘Yen’ threshold function was applied to the green channel image. A binary map was generated and elements with minimum 5 pixels and circularity
0.6 – 1.0 were identified to be lipid droplets and counted. The number of nuclei per image was manually counted and used as an estimate of cell number for lipid count normalization. Analysis was performed in a blinded manner.

**Statistical analysis.** Data are presented as mean ± SEM and statistical analysis was performed using Graphpad Prism V7.0. A description of experimental and biological replicates is provided within the figure legends. No statistical methods were used to determine sample size. For comparison between two groups, a 2-sided Student’s t-test was used. For assessment between two independent variables, two-way ANOVA with Bonferroni multiple comparisons post-hoc test was used. Some datasets required logarithmic transformation to meet equal variance and/or normal distribution statistical assumptions for parametric testing. In datasets where transformation did not achieve equal variances and normal distribution, non-parametric tests were used (Mann Whitney U). Statistical outliers (exceeding 2 standard deviations from the mean) were excluded. A p-value of <0.05 was considered statistically significant.

[1] Bussey CT, Hughes G, Saxena P, Galvin IF, Bunton RW, Noye MK, et al. Chamber-specific changes in calcium-handling proteins in the type 2 diabetic human heart with preserved ejection fraction. Int J Cardiol. 2015;193:53-5.

[2] Shiota M, Printz RL. Diabetes in Zucker diabetic fatty rat. Methods Mol Biol. 2012;933:103-23.

[3] Mellor KM, Varma U, Stapleton D, Delbridge LMD. Cardiomyocyte glycophagy is regulated by insulin and exposure to high extracellular glucose. Am J Physiol Heart Circ Physiol. 2014;306:H1240-5.

[4] Mellor KM, Curl CL, Chandramouli C, Pedrazzini T, Wendt IR, Delbridge LM. Ageing-related cardiomyocyte functional decline is sex and angiotensin II dependent. Age. 2014;36:9630.
