# Supplementary material for: Elevated myocardial fructose and sorbitol levels are associated with diastolic dysfunction in diabetic patients, and cardiomyocyte lipid inclusions in vitro
Source: Nutr Diabetes. 2021 Feb 8;11:8. doi: 10.1038/s41387-021-00150-7 (PMC7870957; doi:10.1038/s41387-021-00150-7)
Supplement: Supplementary file 2 — Supplementary Figures and Table [file 41387_2021_150_MOESM2_ESM.pptx]

## Slide 1
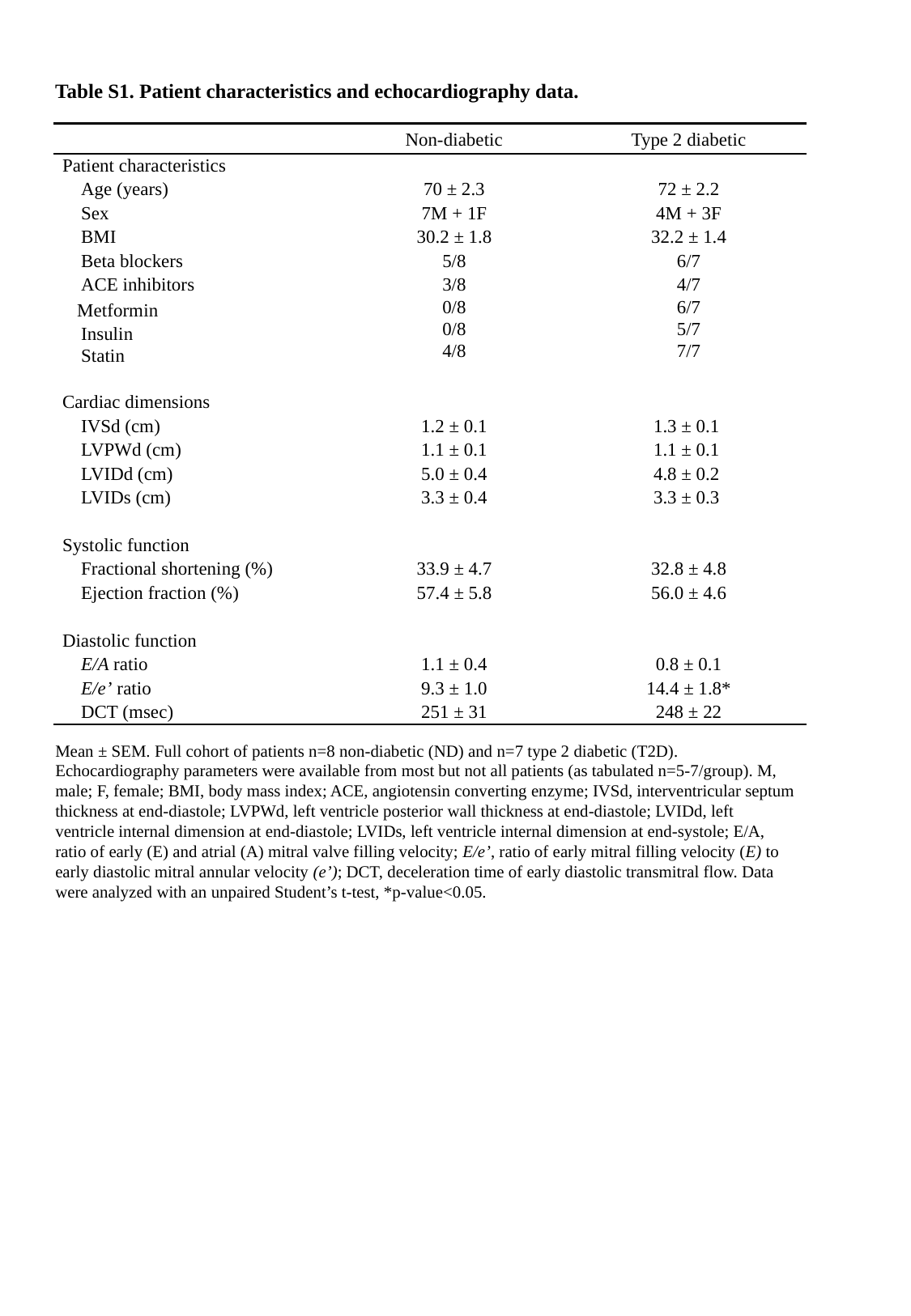

Table S1. Patient characteristics and echocardiography data.
| | Non-diabetic | Type 2 diabetic |
| --- | --- | --- |
| Patient characteristics | | |
| Age (years) | 70 ± 2.3 | 72 ± 2.2 |
| Sex | 7M + 1F | 4M + 3F |
| BMI | 30.2 ± 1.8 | 32.2 ± 1.4 |
| Beta blockers | 5/8 | 6/7 |
| ACE inhibitors Metformin Insulin Statin | 3/8 0/8 0/8 4/8 | 4/7 6/7 5/7 7/7 |
| | | |
| Cardiac dimensions | | |
| IVSd (cm) | 1.2 ± 0.1 | 1.3 ± 0.1 |
| LVPWd (cm) | 1.1 ± 0.1 | 1.1 ± 0.1 |
| LVIDd (cm) | 5.0 ± 0.4 | 4.8 ± 0.2 |
| LVIDs (cm) | 3.3 ± 0.4 | 3.3 ± 0.3 |
| | | |
| Systolic function | | |
| Fractional shortening (%) | 33.9 ± 4.7 | 32.8 ± 4.8 |
| Ejection fraction (%) | 57.4 ± 5.8 | 56.0 ± 4.6 |
| | | |
| Diastolic function | | |
| E/A ratio | 1.1 ± 0.4 | 0.8 ± 0.1 |
| E/e’ ratio | 9.3 ± 1.0 | 14.4 ± 1.8\* |
| DCT (msec) | 251 ± 31 | 248 ± 22 |
Mean ± SEM. Full cohort of patients n=8 non-diabetic (ND) and n=7 type 2 diabetic (T2D). Echocardiography parameters were available from most but not all patients (as tabulated n=5-7/group). M, male; F, female; BMI, body mass index; ACE, angiotensin converting enzyme; IVSd, interventricular septum thickness at end-diastole; LVPWd, left ventricle posterior wall thickness at end-diastole; LVIDd, left ventricle internal dimension at end-diastole; LVIDs, left ventricle internal dimension at end-systole; E/A, ratio of early (E) and atrial (A) mitral valve filling velocity; E/e’, ratio of early mitral filling velocity (E) to early diastolic mitral annular velocity (e’); DCT, deceleration time of early diastolic transmitral flow. Data were analyzed with an unpaired Student’s t-test, *p-value<0.05.

## Slide 2
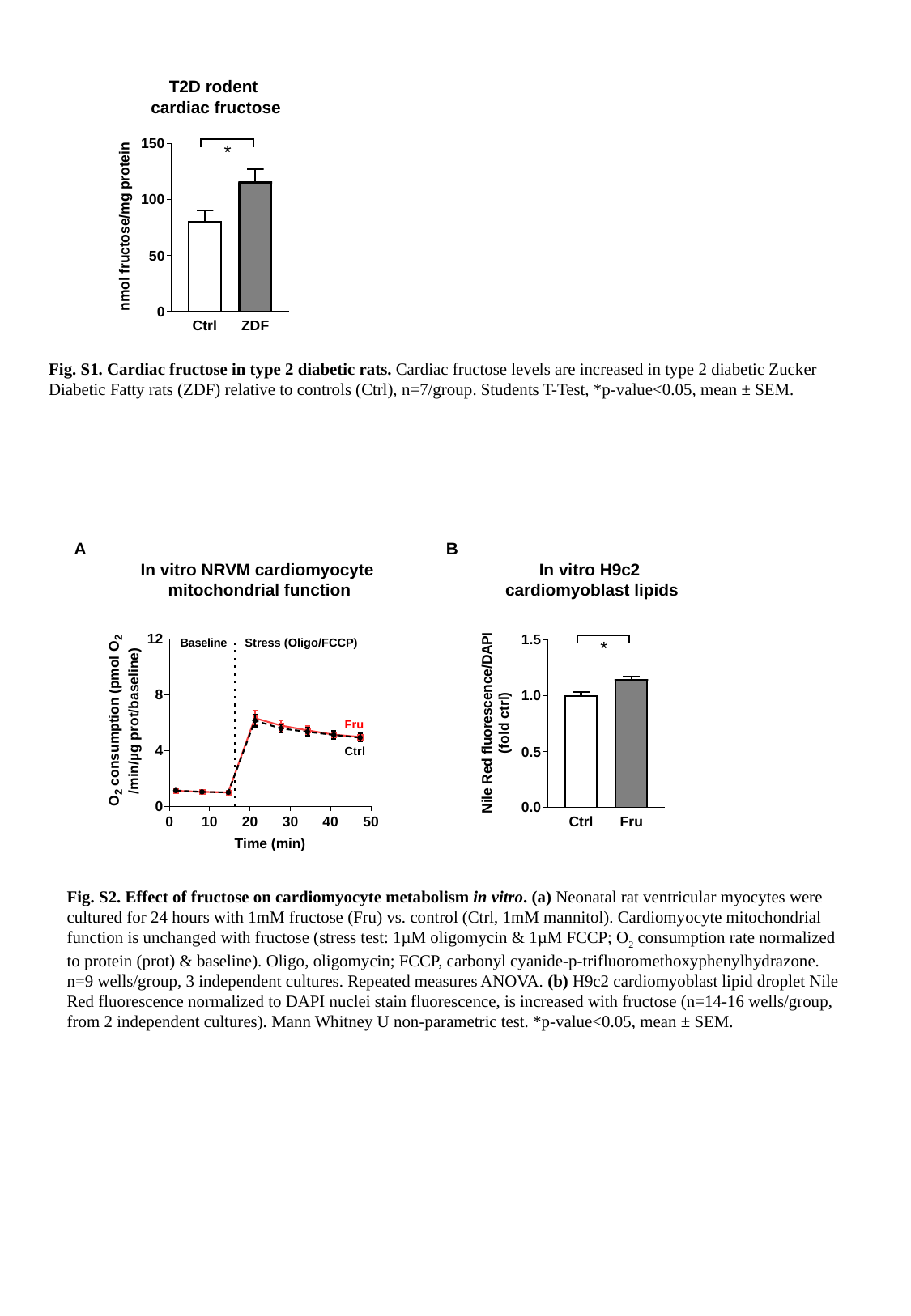

T2D rodent
cardiac fructose
Fig. S1. Cardiac fructose in type 2 diabetic rats. Cardiac fructose levels are increased in type 2 diabetic Zucker Diabetic Fatty rats (ZDF) relative to controls (Ctrl), n=7/group. Students T-Test, *p-value<0.05, mean ± SEM.
A
B
In vitro NRVM cardiomyocyte
mitochondrial function
In vitro H9c2
cardiomyoblast lipids
Fig. S2. Effect of fructose on cardiomyocyte metabolism in vitro. (a) Neonatal rat ventricular myocytes were cultured for 24 hours with 1mM fructose (Fru) vs. control (Ctrl, 1mM mannitol). Cardiomyocyte mitochondrial function is unchanged with fructose (stress test: 1µM oligomycin & 1µM FCCP; O2 consumption rate normalized to protein (prot) & baseline). Oligo, oligomycin; FCCP, carbonyl cyanide-p-trifluoromethoxyphenylhydrazone. n=9 wells/group, 3 independent cultures. Repeated measures ANOVA. (b) H9c2 cardiomyoblast lipid droplet Nile Red fluorescence normalized to DAPI nuclei stain fluorescence, is increased with fructose (n=14-16 wells/group, from 2 independent cultures). Mann Whitney U non-parametric test. *p-value<0.05, mean ± SEM.

## Slide 3
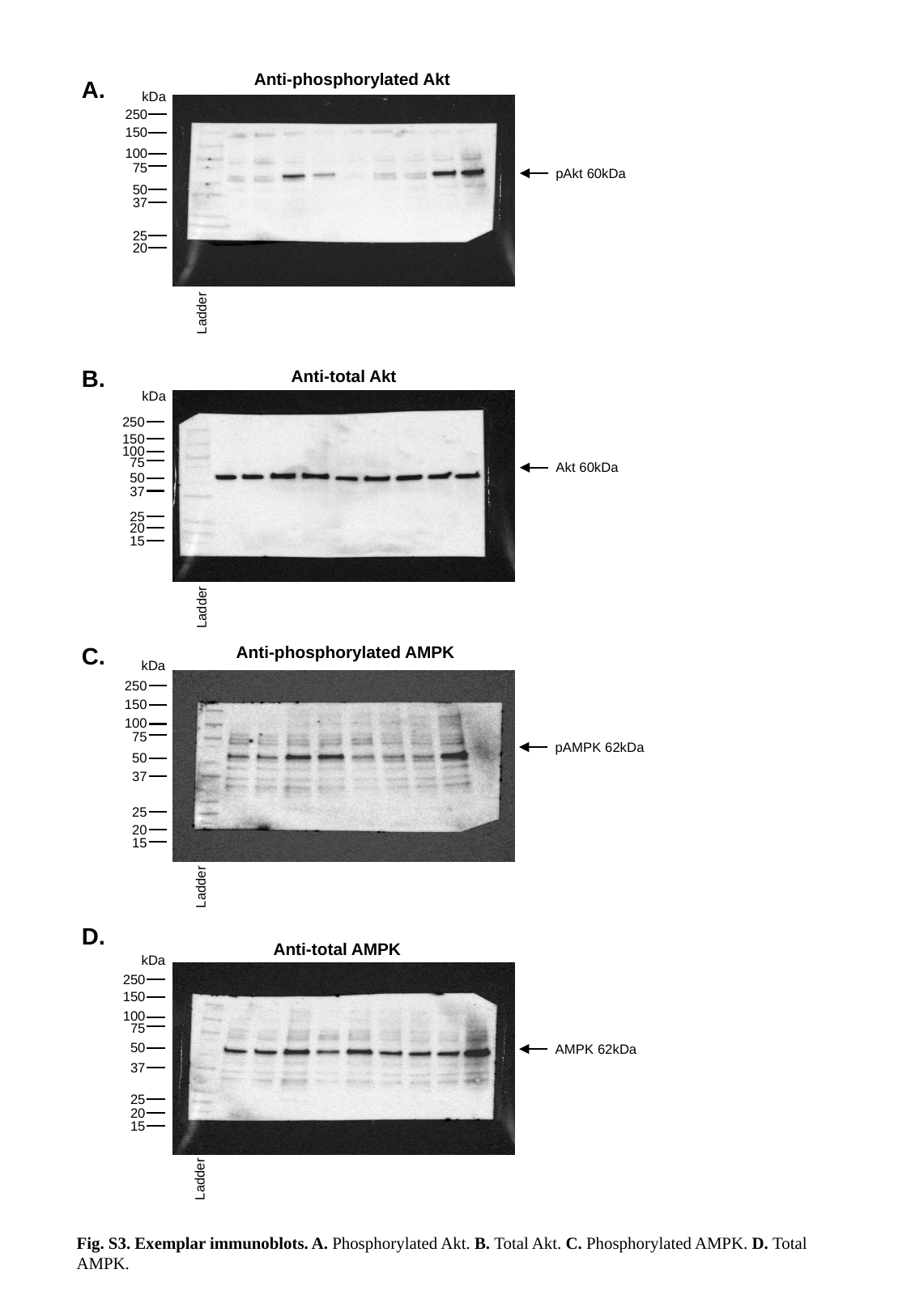

Anti-phosphorylated Akt
A.
kDa
250
150
100
75
pAkt 60kDa
50
37
25
20
Ladder
B.
Anti-total Akt
kDa
250
150
100
75
Akt 60kDa
50
37
25
20
15
Ladder
Anti-phosphorylated AMPK
C.
kDa
250
150
100
75
pAMPK 62kDa
50
37
25
20
15
Ladder
D.
Anti-total AMPK
kDa
250
150
100
75
50
AMPK 62kDa
37
25
20
15
Ladder
Fig. S3. Exemplar immunoblots. A. Phosphorylated Akt. B. Total Akt. C. Phosphorylated AMPK. D. Total AMPK.
